# Testosterone affects female CD4^+^ T cells in healthy individuals and autoimmune liver diseases

**DOI:** 10.1172/jci.insight.184544

**Published:** 2025-04-22

**Authors:** Lara Henze, Nico Will, Dakyung Lee, Victor Haas, Christian Casar, Jasper Meyer, Stephanie Stein, Franziska Mangler, Silja Steinmann, Tobias Poch, Jenny Krause, Johannes Fuss, Johanna Schröder, Alexandra E. Kulle, Paul-Martin Holterhus, Stefan Bonn, Marcus Altfeld, Samuel Huber, Ansgar W. Lohse, Dorothee Schwinge, Christoph Schramm

**Affiliations:** 1 1st Department of Medicine, and; 2 Bioinformatics Core, University Medical Center Hamburg-Eppendorf, Hamburg, Germany.; 3 Institute of Forensic Psychiatry and Sex Research, Center for Translational Neuro and Behavioral Sciences, University of Duisburg-Essen, Essen, Germany.; 4 Institute for Clinical Psychology and Psychotherapy, Department for Psychology, Medical School Hamburg, Hamburg, Germany.; 5 Division of Pediatric Endocrinology and Diabetes, Department of Children and Adolescent Medicine, University Hospital Schleswig-Holstein, Campus Kiel, Kiel, Germany.; 6 Institute of Medical Systems Biology and; 7 Center for Biomedical AI, University Medical Center Hamburg-Eppendorf, Hamburg, Germany.; 8 Research Department Virus Immunology, Leibniz Institute of Virology, Hamburg, Germany.; 9 Hamburg Center for Translational Immunology and; 10 Martin Zeitz Center for Rare Diseases and, University Medical Center Hamburg-Eppendorf, Hamburg, Germany.

**Keywords:** Autoimmunity, Hepatology, Immunology, Autoimmune diseases, Sex hormones, T cells

## Abstract

Autoimmune hepatitis (AIH) and primary biliary cholangitis (PBC) are autoimmune liver diseases with strong female predominance. They are caused by T cell–mediated injury of hepatic parenchymal cells, but the mechanisms underlying this sex bias are unknown. Here, we investigated whether testosterone contributes to T cell activation in women with PBC. Compared with sex- and age-matched healthy controls (*n* = 23), cisgender (cis) women with PBC (*n* = 24) demonstrated decreased testosterone serum levels and proinflammatory CD4^+^ T cell profile in peripheral blood. Testosterone suppressed the expression of TNF and IFN-γ by human CD4^+^ T cells in vitro. In trans men receiving gender-affirming hormone therapy (GAHT) (*n* = 25), testosterone affected CD4^+^ T cell function by inhibiting Th1 and Th17 differentiation and by supporting the differentiation into regulatory Treg. Mechanistically, we provide evidence for a direct effect of testosterone on T cells using mice with T cell–specific deletion of the cytosolic androgen receptor. Supporting a role for testosterone in autoimmune liver disease, we observed an improved disease course and profound changes in T cell states in a trans man with AIH/primary sclerosing cholangitis (PSC) variant syndrome receiving GAHT. We here report a direct effect of testosterone on CD4^+^ T cells that may contribute to future personalized treatment strategies.

## Abstract 2

We here report a direct effect of testosterone on CD4^+^ T cells that may contribute to future personalized treatment strategies.

## Graphical abstract

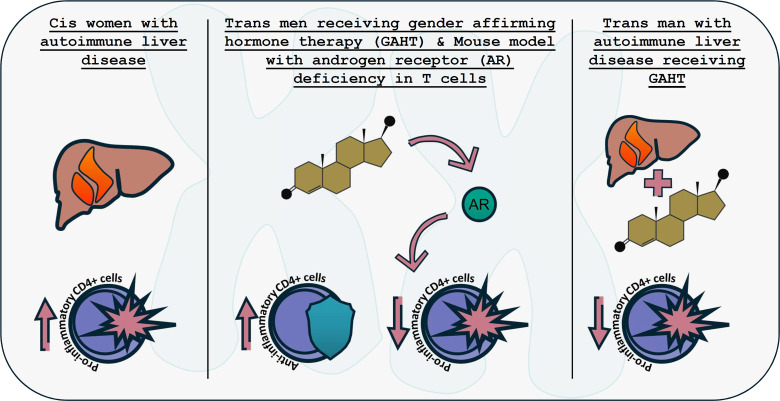

## Introduction

There is increasing interest in sex- and gender-related differences in immune responses. Whereas females show better control of viral infections, they are generally more prone to develop autoimmune diseases (1–4). This partly relates to sex, as determined by the sex chromosomes, for example via selective X-chromosomal inactivation (5, 6). However, sex hormones also directly or indirectly contribute to the regulation of the immune system (7). A substantial role in modulating the immune system has been described for 17-β estradiol (E2). The effects of E2 on immune cells varies depending on factors such as the concentration, the specific immune cell type, and the context of the immune response. Low concentrations of E2 have been associated with proinflammatory immune responses, whereas high concentrations of estrogens have been reported to be antiinflammatory and to augment Th2 responses and humoral immunity (8). Immunomodulatory effects have also been demonstrated for androgens, and testosterone has been shown to suppress the activity of immune cells, leading to immunosuppression. Thus, testosterone has been demonstrated to reduce proinflammatory responses of macrophages and to suppress extracellular signal–regulated kinases and leukotriene formation in neutrophils (9, 10). It has also been shown that healthy female mice have higher numbers of ILC2 cells in multiple tissues and that they can be modulated by testosterone (11). In murine-derived splenocytes, testosterone reduced the production of proinflammatory cytokines such as IL-1, IL-6, and TNF, while potentially increasing the production of antiinflammatory cytokines such as IL-10 in antigen-specific stimulated CD4^+^ T cells (12). Little is known about the effects of androgens on T cells in humans, but lower CD8^+^ T cell and higher CD4^+^ T cell frequencies, and specifically a higher frequency of circulating Treg, have been described in cisgender (cis) women compared with cis men (13, 14). Moreover, in serum from men with androgen deficiency, higher concentrations of proinflammatory cytokines and higher CD4^+^/CD8^+^ T cell ratios were reported (15–17). Notably, lower serum levels of testosterone were reported for autoimmune diseases, including systemic lupus erythematosus (SLE), multiple sclerosis (MS), and rheumatoid arthritis (RA) (18, 19). So far, it is unclear whether those effects might contribute to T cell regulation in autoimmune diseases.

In this study, we sought to investigate the effects of testosterone on T cell function in healthy humans and humans affected by primary biliary cholangitis (PBC), an autoimmune liver disease (AILD) with a particularly high female predominance (female/male ratio of 9:1) (20, 21). We found that testosterone levels associated with proinflammatory T cell phenotype in women with PBC affect T cell differentiation in trans men and that testosterone exerts direct effects on murine T cells, as shown by using mice with T cell–specific androgen receptor (AR) deficiency. Our data on a trans man with AILD provide evidence that understanding how androgen signaling modulates autoimmune disease might reveal treatment targets in the future.

## Results

### Proinflammatory immune cell phenotype in cis women with PBC.

**Figure 1:**
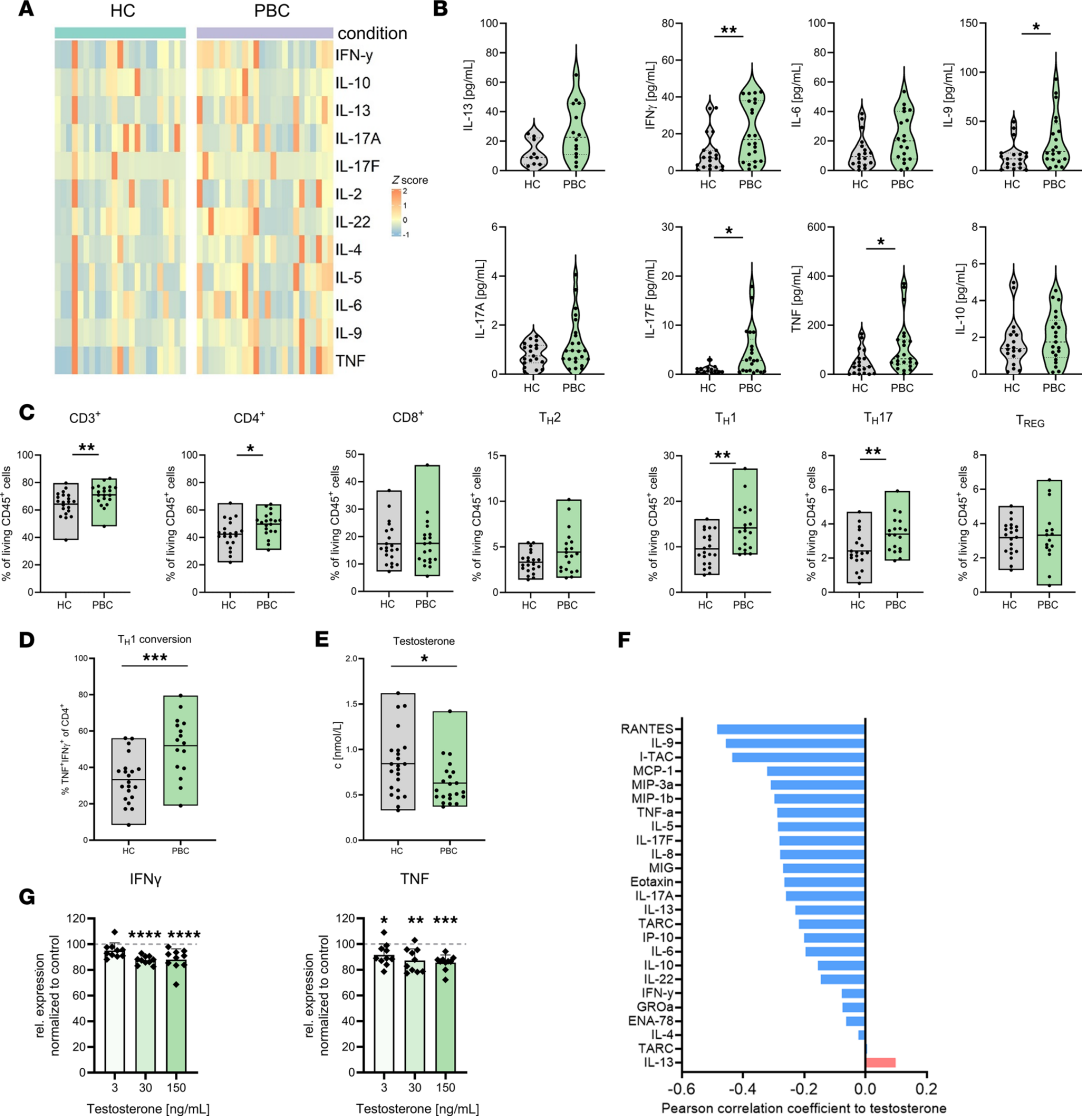
Immune profile of cis women with PBC. (**A** and **B**) Serum cytokine levels determined by flow cytometry–based LEGENDplex analysis from cis women with PBC (*n* = 24) compared with age- and sex-matched healthy controls (*n* = 23). (**C**) Frequencies of CD3^+^, CD4^+^ CD8^+^, Th2, Th1, Th17, and Treg from cis women with PBC (*n* = 17–21) compared with age- and sex-matched healthy controls (*n* = 21–22) were analyzed by flow cytometry. (**D**) Th1 differentiation rate was increased in CD4^+^ T cells derived from cis women with PBC (*n* = 16) compared with age- and sex-matched healthy controls (*n* = 21). (**E**) Serum testosterone levels of cis women with PBC (*n* = 27) compared with age- and sex-matched healthy controls (*n* = 23) determined by LC-MS/MS analysis. (**F**) Correlation analysis of serum testosterone levels and serum cytokine profiles determined using flow cytometry–based LEGENDplex analysis from cis women with PBC (*n* = 27). (**G**) Decreased concentrations of IFN-γ and TNF in supernatant of T cell receptor–stimulated T cells derived from healthy cis women in the presence of testosterone. Cytokine secretion was analyzed in supernatants using ELISA, and statistical significance was determined via 1-way ANOVA (**G**). Statistical analysis was performed using Mann-Whitney *U* test (**B**–**E**). *P ≤ 0.05; **P ≤ 0.01; ***P ≤ 0.001, ****P ≤ 0.0001.

The reasons for the female predominance in PBC are largely unknown. In order to investigate the underlying mechanisms, we first aimed to deeply characterize the immunological phenotype of cis women with PBC (*n* = 24) compared with age- and sex-matched healthy controls (*n* = 23). To that end, we determined serum cytokine levels using LEGENDplex analysis and performed multicolor flow cytometry–based immunophenotyping. We observed an increased proinflammatory serum cytokine profile in cis women with PBC compared with age- and sex-matched healthy controls (Figure 1A), with the most pronounced differences for IL-9, TNF, IFN-γ, and IL-17F (Figure 1B). Peripheral blood mononuclear cells (PBMCs) showed increased frequencies of CD3^+^, CD4^+^, Th1, and Th17 cells and an increased in vitro differentiation capacity of naive CD4^+^ T cells toward Th1 cells (Figure 1, C and D). To investigate which of the sex hormones might associate with the proinflammatory state in PBC, we next determined the serum concentrations of 15 steroid hormone levels using liquid chromatography with tandem mass spectrometry–based (LC-MS/MS–based) quantitative analysis. We found significantly lower testosterone levels in cis women with PBC compared with age- and sex-matched healthy controls (Figure 1E and Supplemental Figure 1; supplemental material available online with this article; https://doi.org/10.1172/jci.insight.184544DS1). Testosterone levels negatively correlated with serum cytokines, including proinflammatory cytokines IFN-γ and IL-6, which had previously been shown to be elevated in PBC (Figure 1F) (22). To support the hypothesis that testosterone affects the human T cell phenotype, we performed in vitro stimulation experiments of TCR-activated T cells isolated from healthy female donors in the presence or absence of testosterone and showed that testosterone led to a significantly reduced secretion of the proinflammatory cytokines IFN-γ and TNF (Figure 1G). Overall, these data support the notion that testosterone reduces the activation of human CD4^+^ T cells, which is in line with our previous findings on the suppressive effects of testosterone on CD4^+^ T cells in a mouse model of cholangitis (20).

### Gender-affirming hormone therapy has subtle effects on T cell transcriptomes in healthy trans men.

**Figure 2:**
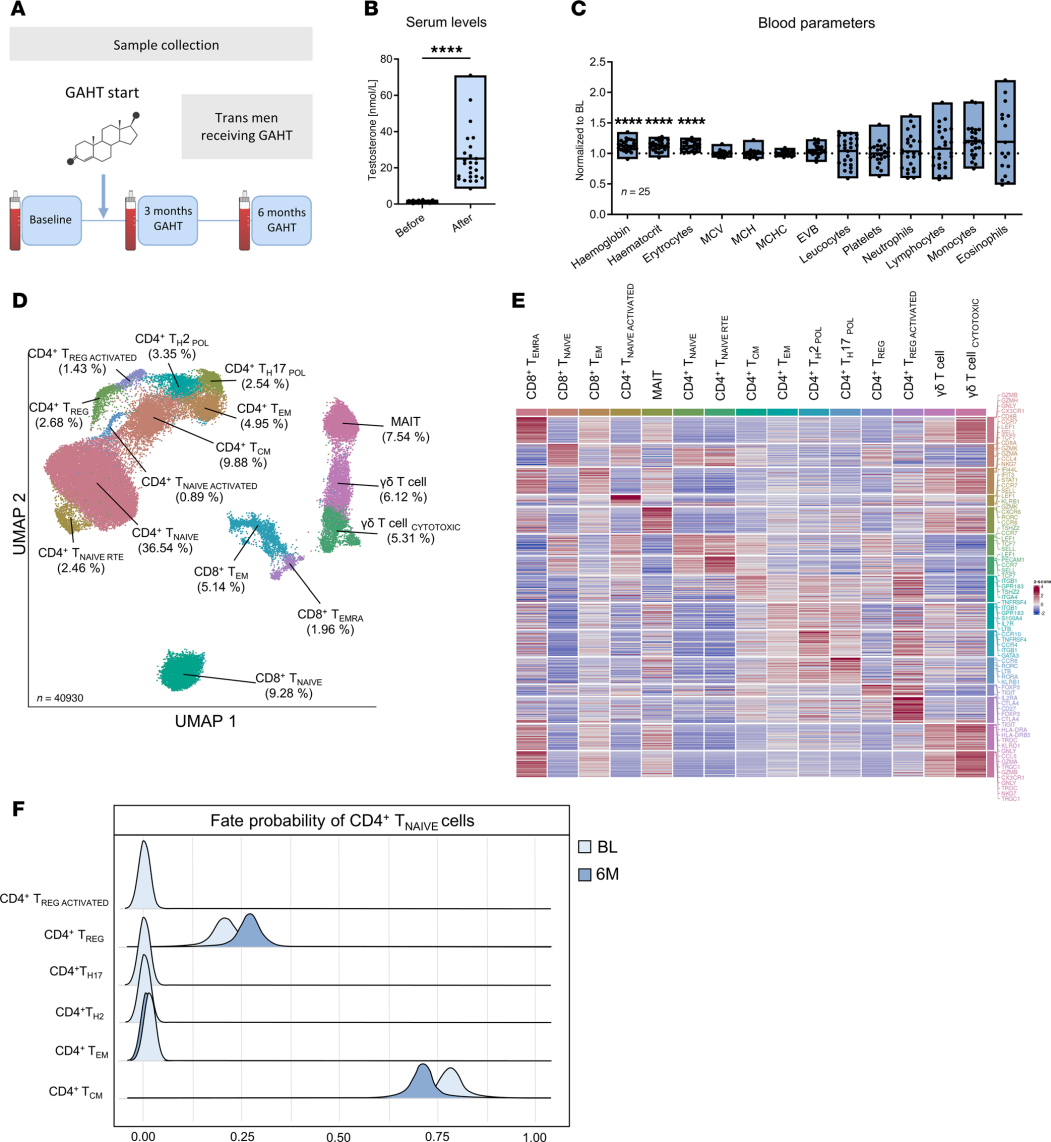
Gender-affirming hormone therapy (GAHT) with a high dose of testosterone alters CD4^+^ T cell phenotype in trans men. (**A**) Graphical abstract of the cohort included. (**B**) Serum testosterone levels of trans men receiving GAHT at baseline (BL) before and 6 months (6M) after therapy start with testosterone (*n* = 25). (**C**) Blood parameters from trans men at 6M of GAHT normalized to intrapersonal baseline values (*n* = 25). (**D**) UMAP resembling 40,930 peripheral blood T cells of trans men at BL and the 6M time point (*n* = 4). Cells were subdivided into 15 clusters by Seurat: 6 clusters predominantly expressed CD8^+^ T cell markers (γδ T cells, γδ T cells_CYTOTOXIC_, MAIT, CD8^+^ T_NAIVE_, CD8^+^ Temra, and CD8^+^ Tem) and 9 clusters expressed CD4^+^ T cell markers (CD4^+^ T_NAIVE_, CD4^+^ T_NAIVE_ _RTE_, CD4^+^ T_NAIVE_ _ACTIVATED_, CD4^+^ Tcm, CD4^+^ Treg, CD4^+^ Treg _ACTIVATED_, CD4^+^ Tem, CD4^+^ Th17_POL_, and CD4^+^ Th2_POL_). (**E**) Heatmap showing signature differentially expressed genes (DEG) of each cluster. (**F**) Fate probability analysis of CD4^+^_NAIVE_ T cells toward different endpoints is displayed. After 6 months of GAHT (dark blue), the fate probability of naive T cells was increased toward the Treg endpoint and decreased toward Tcm compared with naive CD4^+^ T cells before therapy (light blue). Data are shown as mean ± SD. Statistical analysis was performed using 1-way ANOVA test. ****P ≤ 0.0001.

It is challenging to study the effects of testosterone on human T cell function in vivo. To do so, we prospectively collected blood from healthy trans men receiving gender-affirming hormone therapy (GAHT) with testosterone (Figure 2A). Successful treatment with testosterone was confirmed by measuring significantly increased serum testosterone levels 3 months (3M) and 6M after the start of GAHT compared with baseline levels (BL) (Figure 2B). Validating the biological effects of testosterone on hematopoietic cells and in accordance with previously published data (22), we observed significant increases in hemoglobin, hematocrit, and RBC count (Figure 2C). In order to investigate the effect of testosterone on human T cells in vivo in more detail, we performed Cellular Indexing of Transcriptomes and Epitopes by Sequencing (CITE-Seq) on PBMCs at BL and the 6M time point. T cells sorted by FACS from 4 trans men were used for sequencing, combining 40,930 cells that were assigned to 15 T cell clusters by differentially expressed genes and surface markers (Figure 2, D and E). Six clusters were identified as CD8^+^ T cells, including γδ T, γδ T_CYTOTOXIC_, MAIT, CD8^+^ T_NAIVE_, CD8^+^ effector memory T (Tem), and CD8^+^ effector memory reexpressing CD45RA (Temra) cells. Guided by our previous results from cis women with PBC, we focused on the 9 clusters of CD4^+^ T cells with the highest number of cells, representing naive CD4^+^ T cells (CD4^+^ T_NAIVE_) expressing CCR7, LEF1, and SELL. Two further distinct naive CD4^+^ clusters consisted of recent thymic emigrant (RTE) T cells (CD4^+^ T_NAIVE_ _RTE_) expressing PECAM1 and activated naive CD4^+^ T cells (CD4^+^ T_NAIVE_ _ACTIVATED_) expressing multiple IFN-induced genes and STAT1, besides the typical naive markers. The second largest CD4^+^ cluster was identified as central memory T cells (CD4^+^ Tcm) expressing ITGB1 and GPR183, paired with antibody-derived tag (ADT) signals for CD62L. Furthermore, we identified 2 clusters of CD4^+^ Treg (CD4^+^ Treg and CD4^+^ Treg _ACTIVATED_) expressing FOXP3, IL-2RA, and CTLA4. Notably, CD4^+^ Treg _ACTIVATED_ were defined by the expression of the additional activation markers HLA-DRA and HLA-DRB5 as well as by higher expression of CD25. Other CD4^+^ T cell populations were effector memory cells (CD4^+^ Tem), characterized by low CCR7 and SELL but high expression of S100A4; Th17-polarized cells (CD4^+^ Th17_POL_), which express CCR6, RORC, KLRB1, and RORA; and Th2-polarized cells (CD4^+^ Th2_POL_), expressing GATA3, CCR4, and CCR10. Interestingly, we could only identify minor changes in differentially expressed genes (DEG) within CD4^+^ T cell clusters under testosterone treatment, as defined by a combination of a 25% difference in expression and statistical significance. We further assessed the differentiation potential of naive CD4^+^ T cells into other subclusters and performed propensity analysis using CellRank (23). We observed that, following in vivo exposure to a high dose of testosterone for a duration of 6M, naive CD4^+^ T cells exhibited a transcriptional potential to differentiate into Treg and a reduced potential to differentiate into CD4^+^ Tcm and CD4^+^ Tem (Figure 2F).

### GAHT results in an antiinflammatory shift of CD4^+^ T cells in trans men.

**Figure 3:**
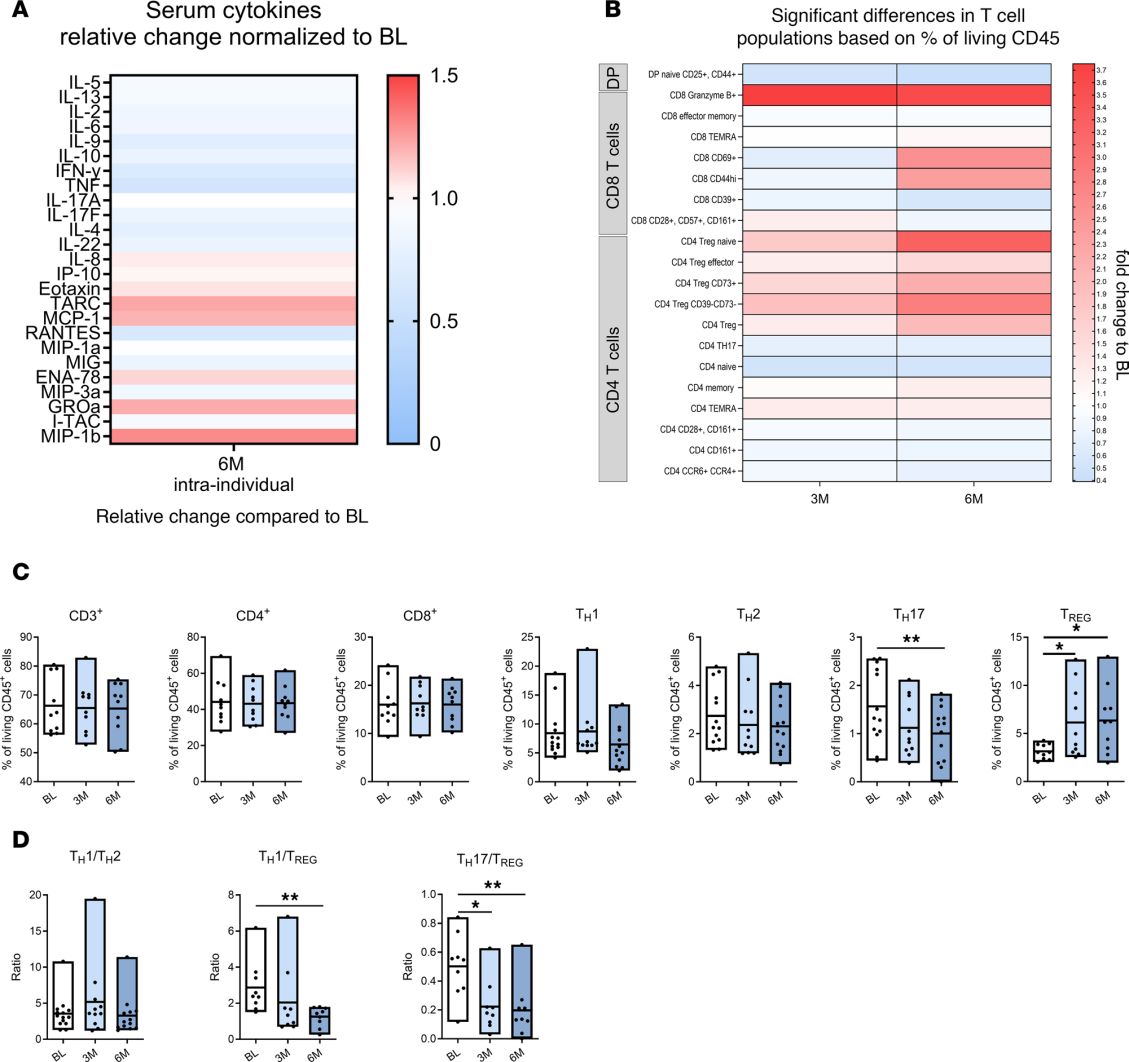
Shift toward antiinflammatory immune cell subsets in trans men receiving GAHT. (**A**) Heatmap of intraindividual serum cytokine changes in trans men (*n* = 25) 6M after GAHT normalized to baseline before therapy start. Serum cytokine levels were determined by flow cytometry–based LEGENDplex analysis. (**B**) Heatmap of T cell populations identified as significantly different by flow cytometry–based immunophenotyping 6M after therapy start (*n* = 22). Red indicates an increase and blue indicates a decrease compared with baseline samples. (**C**) Frequencies of CD3^+^, CD4^+^, CD8^+^, Th17, Th1, Th2, and Treg from trans men at BL, 3M, and 6M time points analyzed by flow cytometry. (**D**) Shift in T cell phenotypes toward antiinflammatory immune cell subsets. Data are shown as mean ± SD. Statistical analysis was performed using a 1-way ANOVA with correction for multiple comparisons. *P ≤ 0.05; **P ≤ 0.01.

We next aimed to investigate potential effects of testosterone on T cells by analyzing their protein expression. We therefore determined the effect of GAHT on serum cytokines and immune cells by performing LEGENDplex analysis of serum and flow cytometry–based immunophenotyping in PBMCs obtained from trans men (*n* = 25) at BL and after 6M of high-dose testosterone treatment. Testosterone therapy resulted in a broad reduction of proinflammatory cytokine serum levels and an increase in levels of chemokines such as CXCL1 (GROa), CCL2 (MCP-1), and CCL4 (MIP-1b) (Figure 3A). Flow cytometry data obtained from PBMCs were analyzed using SPADE-VizR and were verified by manual gating (24) (Figure 3B). Whereas frequencies of granzyme B–expressing CD8^+^ T cells seemed to increase upon testosterone treatment, CD4^+^ T cells showed shifts to a more suppressive phenotype, with reduced frequencies of Th17 cells and significantly increased Treg frequencies (Figure 3C). Ratios of Th17 or Th1 cells to Treg were significantly decreased upon testosterone treatment (Figure 3D). These data support the notion that testosterone induces an antiinflammatory shift in human CD4^+^ T cells not only in vitro but also in vivo.

### Testosterone directly acts on CD4^+^ T cells via the cytosolic AR receptor.

**Figure 4:**
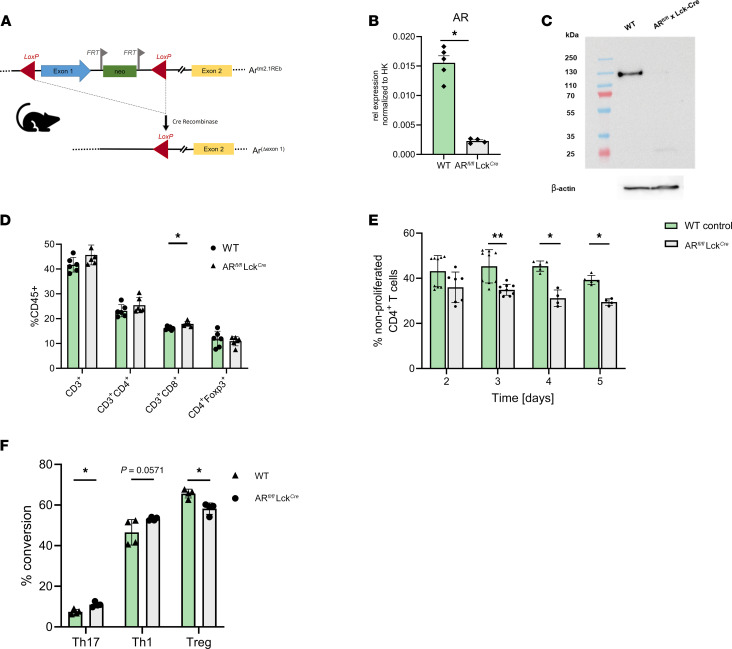
Testosterone directly acts on murine T cells via the cytosolic AR. (**A**) Schematic of breeding strategy for mice lacking AR signaling in mature T cells. (**B** and **C**) qRNA expression and protein expression of AR in CD4^+^ T cells isolated from AR^fl/fl^ Lck^Cre^ mice (green stroke pattern) compared with WT littermates (green). (**D**) Immune cell phenotyping in AR^fl/fl^ Lck^Cre^ female mice (green stroke pattern) compared with WT female littermates (green) (*n* = 5). (**E** and **F**) In vitro proliferation assay and in vitro differentiation assay of CD4^+^ T cells isolated from AR^fl/fl^ Lck^Cre^ female mice (green stroke pattern) compared with WT female littermates (green). Representative figures of 3 independent experiments and mean ± SD are shown. Statistical analysis was performed using Mann-Whitney *U* test. *P ≤ 0.05; **P ≤ 0.01.

To investigate whether testosterone directly affects T cells and whether these effects are mediated via the classical cytosolic AR, we generated mice with a conditional KO of AR in T cells by crossing AR^fl/fl^ mice with Lck^Cre^ transgenic mice that contain a Cre-recombinase gene driven by the distal promoter of the lymphocyte protein tyrosine kinase (Lck) gene to avoid thymic deletion (Figure 4A) (25). Absence of AR expression was confirmed on mRNA and protein level in female CD4^+^ T cells compared with cells derived from littermate WT controls (Figure 4, B and C). We found similar frequencies of splenic CD3^+^, CD4^+^, and CD8^+^ T cell populations in female mice lacking the AR in T cells (Figure 4D). Briefly, upon T cell receptor–specific stimulation in vitro, we observed an enhanced proliferation rate of CD4^+^ T cells isolated from spleen of mice lacking AR signaling compared with WT littermate controls (Figure 4E). To prove that AR signaling in T cells affects CD4^+^ T cell differentiation, we performed in vitro differentiation assays and observed significantly increased Th17 differentiation and decreased differentiation toward Treg (Figure 4F). Taken together, these data confirm a direct and AR-mediated effect of testosterone on murine CD4^+^ T cell differentiation and activation. To further validate these findings at the level of cytokine release, we performed ex vivo stimulation experiments with CD4^+^ T cells derived from WT and AR-deficient mice. Specifically, ELISA analysis revealed significantly increased concentrations of IL-17A and IL-22 in Th17 differentiation conditions and increased IFN-γ concentrations in Th1 differentiation conditions in AR-deficient mice compared with WT controls (Supplemental Figure 5). These results align with the observed differentiation rates depicted in Figure 4F and further support the role of AR-mediated testosterone signaling in modulating murine T cell differentiation.

### Improved clinical disease course observed in a trans man with AILD during GAHT.

**Figure 5:**
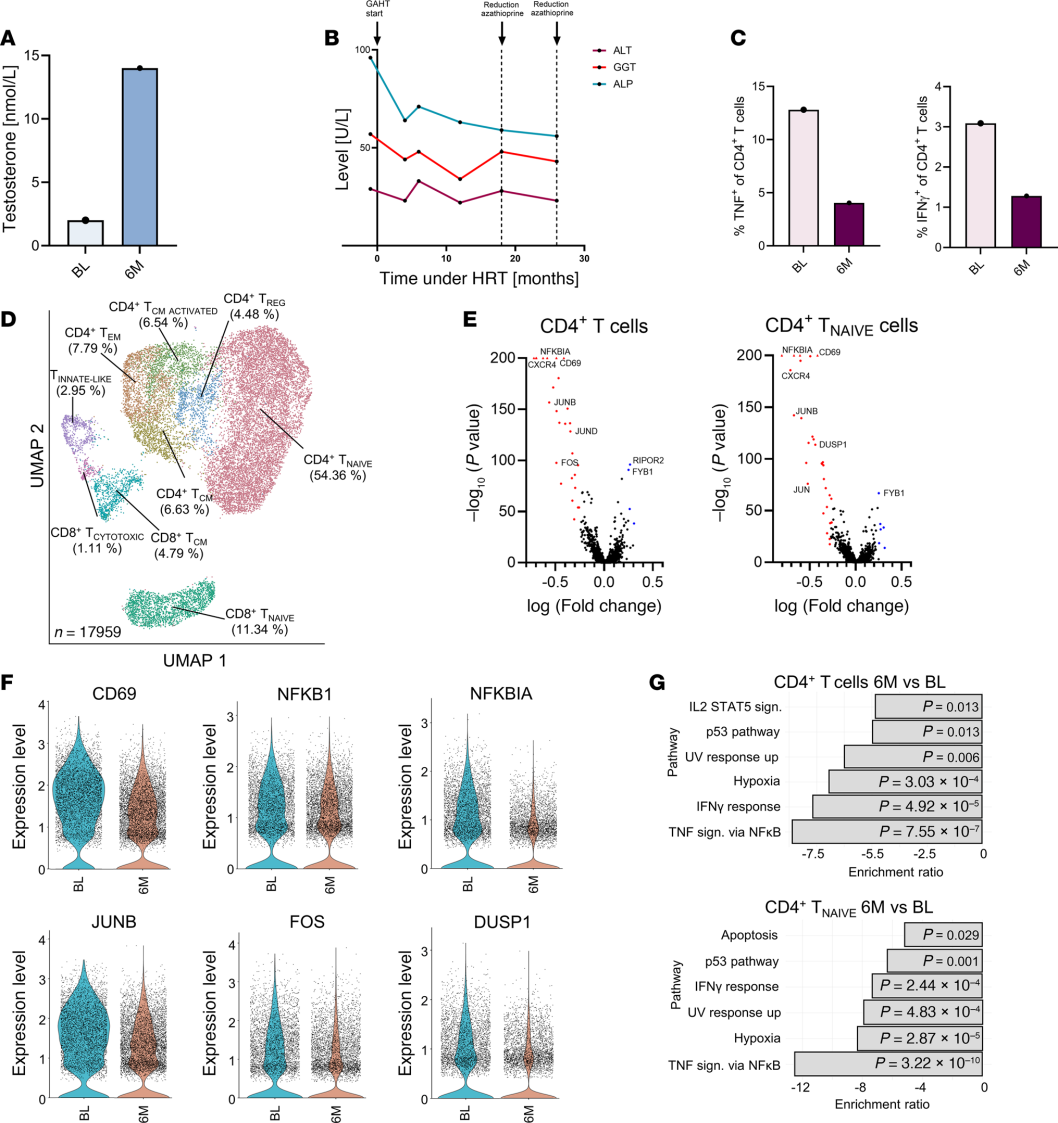
Gender-affirming hormone therapy improves autoimmune liver disease in a trans man. Single-case study of a trans man with AIH/PSC variant syndrome receiving GAHT. (**A**) Serum testosterone levels measured using LC-MS/MS analysis. (**B**) Clinical parameters determined by serum transaminases and IgG levels before and during testosterone therapy. (**C**) TNF- and IFN-γ–expressing CD4^+^ T cells in blood before and after 6M of GAHT. (**D**) UMAP reflecting 17,959 peripheral blood CD3^+^ T cells at baseline (BL) and 6M after GAHT showing 9 distinct T cell clusters (5 CD4^+^ and 4 predominantly CD8^+^). (**E**) Volcano plots of differentially expressed genes (DEGs) of total CD4^+^ and CD4^+^ T_NAIVE_ cells. (**F**) Violin plots of the gene expression of CD69, NFKB1, NFKBIA, JUNB, FOS, and DUSP1 at BL and 6M in the CD4^+^ T_NAIVE_ cluster. (**G**) Overrepresentation analysis (ORA) of significantly changed pathways according to the Hallmark database in CD4^+^ and CD4^+^ T_NAIVE_ cells. Indicated *P* values were corrected for multiple comparisons.

So far, there are no reports on how testosterone therapy might affect the activity and course of AILD. Here, we report our findings from examining a unique case of a trans man with autoimmune hepatitis (AIH)/primary sclerosing cholangitis (PSC) variant syndrome undergoing GAHT. Successful testosterone therapy was confirmed by measuring serum testosterone levels before and during treatment (Figure 5A). Interestingly, during high-dose testosterone treatment, we observed an improved clinical disease course defined by a reduction in serum levels of γ glutamyltransferase (GGT) and alkaline phosphatase (ALP) as markers of biliary injury and cholestasis. Although alanine aminotransferase (ALT) levels fluctuated over time, they at least remained stable during the course of GAHT despite reduced intensity of immunosuppressive therapy; azathioprine could be reduced from 125 to 25 mg per day during GAHT (Figure 5B). In addition, liver stiffness as a marker of fibrosis stage tended to decline along with the biochemical improvements (Supplemental Figure 2A). The frequency of TNF- and IFN-γ–expressing CD4^+^ T cells in peripheral blood had declined after 6M of GAHT (Figure 5C). To identify changes in immune cell composition in more detail, we performed CITE-Seq analysis on presorted CD3^+^ T cells from peripheral blood at BL and 6M after the start of GAHT. In total, 17,959 cells were included. We were able to distinguish 9 T cell clusters according to the expression of surface markers and signature genes (Supplemental Figure 2B). Briefly, 5 CD4^+^ T cell clusters were identified, including CD4^+^ Tem, CD4^+^ Tcm, CD4^+^ Tcm _ACTIVATED_, CD4^+^ T_NAIVE_, and Treg. As expected, CD4^+^ T_NAIVE_ cells represented the biggest cluster while Treg represented the smallest CD4^+^ cluster in this dataset. For CD8^+^ T cells, we identified 4 different clusters, with the biggest cluster being CD8^+^ T_NAIVE_ followed by CD8^+^ Tem, innate-like T cells (T_INNATE_ _LIKE_), and CD8^+^ T_CYTOTOXIC_ (Figure 5D). To determine differences between BL and 6M of GAHT, we analyzed DEGs between the time points within all clusters and investigated the most notable changes within the entire CD4^+^ T cell and CD4^+^ T_NAIVE_ cell clusters (Figure 5E). GAHT was associated with decreased expression of the markers CD69 and CXCR4 and a lower expression of CD83, TSC22D3, and RGS1 in the entire CD4^+^ and the CD4^+^ T_NAIVE_ cell clusters. This is of interest since the genes CD69 and CXCR4 are involved in T cell activation and homing toward the liver and the biliary epithelium (26) and the expression of CD83, TSC22D3, and RGS1 is associated with a precursor state of exhausted T cells (27, 28). Strikingly, GAHT was associated with a significantly reduced expression of multiple genes related to T cell activation and Th1/Th17 differentiation (JUN, JUNB, PIK3IP1, DUSP1/2) within the CD4^+^ and CD4^+^ T_NAIVE_ clusters (29–31) as well as numerous genes linked to the NF-κB signaling pathway. Notably, the marked effect of testosterone on CD4^+^ T_NAIVE_ cells (Figure 5F) is in line with our findings described above. We next performed overrepresentation analysis (ORA) on the DEGs and found downregulation of TNF signaling through the NF-κB and IFN-γ response in CD4^+^ and CD4^+^ T_NAIVE_ cells (Figure 5G). Thus, this in-depth analysis of GAHT in a trans man with AILD revealed potential antiinflammatory effects of testosterone not only on T cell subsets in vivo but also on biomarkers of disease activity along a reduction in immunosuppressive treatment intensity.

## Discussion

Cis women have been reported to have stronger immune responses than cis men. This can confer advantages in the defense against infections, but it may also contribute to a higher susceptibility to autoimmune diseases (32–34). Despite well-established differences in incidence and disease course of autoimmune diseases between cis women and cis men, the underlying biological mechanisms remain incompletely understood. In this study, we report an effect of testosterone on human CD4^+^ T cells, shifting them toward an antiinflammatory cell state.

Testosterone is the primary male sex hormone, but it is also produced by females, albeit in substantially lower quantities than males. Notably, cis women of all ages have testosterone serum concentrations in the nanomolar range, which is higher than the picomolar concentrations of the primary female sex hormone estradiol (35). Published data point to testosterone being a modulator of immune responses, but most of the studies were performed in mouse models or using human cells in vitro. Thus, testosterone has been described to reduce the expression of the proinflammatory cytokine IFN-γ and the transcription factor T-bet (TBX21), which is important for the differentiation into Th1 subsets, and to increase the expression of IL-10 in murine CD4^+^ T cells (12). We have previously demonstrated that testosterone was able to modulate biliary injury via T cells in vivo. Briefly, in a T cell–driven mouse model of antigen-specific autoimmune cholangitis, a strong sex dimorphism was observed, with female mice developing severe cholangitis, whereas male mice were resistant to cholangitis induction (36). In this model, we demonstrated that testosterone treatment was sufficient to completely suppress liver inflammation in female mice, significantly reducing recruitment and IL-17 production of CD4^+^ T cells. A recent study investigated the effect of androgens on cell populations in various mouse organs. This study highlighted that hepatic T cells are among the cells with the largest transcriptional differences upon androgen depletion and supplementation (11). These data provide evidence that androgens might contribute to sex-related differences in T cell phenotype and the cellular composition of the liver.

Although PBC and AIH are much more common in females, the mechanisms underlying the sexual dimorphism in AILD are largely unknown and have not yet been investigated in detail. Sex hormones have been hypothesized to play a role in causing the diseases, and the incidence of PBC and AIH peaking around menopause indeed points to a contribution of sex hormones (20, 37). In this context it is important to note that sex hormone levels vary throughout life, and hormonal changes are a natural part of aging, puberty, the menstrual cycle, pregnancy, and menopause (38). Testosterone is produced in the ovaries of females during their reproductive years as well as by peripheral conversion of androstenedione and dehydroepiandrosterone (DHEA) in the adrenal glands. With aging, there is an overall decrease in androgen production due to age-related decline of ovarian and adrenal function. However, this seems to be unrelated to menopause development (39–42). We here report reduced serum testosterone levels in cis women with PBC compared with healthy age- and sex- matched controls. This is in accordance with reports from other autoimmune diseases, including SLE, MS, and RA, in which lower serum levels of testosterone have previously been reported (18, 19). Furthermore, testosterone serum levels negatively correlated with concentrations of proinflammatory cytokines IFN-γ and TNF, which are related to T cell activation (Figure 1). In addition to the observed correlations between testosterone levels and serum cytokine profiles, further analyses were performed to evaluate potential relationships with other clinical parameters (Supplemental Figure 3, A–C, and E). There was no correlation between serum testosterone levels and liver stiffness, age, or BMI, suggesting that the observed immune phenotypes are unlikely to be confounded by these variables. Since T cells play an important role in the pathogenesis of AIH and PBC, we further investigated the effect of testosterone on human and murine T cells. While we observed only subtle differences in the CD4^+^ T cell state in healthy trans men on a transcriptional level, a shift of CD4^+^ T cells toward an antiinflammatory phenotype was observed at the protein level (Figures 2 and 3). Importantly, the significantly increased Treg frequencies during GAHT are consistent with data from Robinson et al., who found a significant increase in Treg in both cis men and trans men compared with cis women (13). In order to prove that androgens may directly act on T cells, we generated mice lacking the AR. In steady state and healthy mice, we observed that AR deficiency in mature T lymphocytes resulted in increased CD4^+^ T cell proliferation and a shift toward differentiation into a proinflammatory T cell phenotype (Figure 4). Moreover, ex vivo cytokine data confirm increased IL-17A, IL-22, and IFN-γ levels in AR-deficient mice under Th17 and Th1 differentiation conditions, respectively, validating the effect of AR signaling on T cell cytokine production on the protein level. However, while these data highlight the direct suppressive effect of testosterone on T cells, it is important to acknowledge that in vitro experiments, including both murine and human studies such as in Figure 1G, are limited in their ability to fully capture the complexity of in vivo immune responses. The interaction of T cells with other immune and nonimmune cell types, as well as the influence of the broader immune microenvironment, may modify or amplify the effects observed in isolated T cells. Thus, they should be interpreted with caution. A combination of AR-deficient T cell models with models for AILD-like conditions, such as the K14-OVAp model of T cell–mediated autoimmune cholangitis (36), could be investigated to further explore AR signaling in disease settings in the future.

Interestingly, a recent study by Stülb et al. investigating acetaminophen-induced liver injury in mice highlighted that IL-22, a key cytokine in liver regeneration, is produced at higher levels in female mice compared with males. Furthermore, they demonstrated that testosterone reduced IL-22 production in female, but not male, splenocytes (43). This suggests that testosterone modulates T cell responses in a sex-dependent manner, potentially influencing the course of liver injury and immune-mediated diseases. Furthermore, our ex vivo stimulation experiments (Supplemental Figure 4B) revealed tendencies for decreased release of signature Th1 and Th17 cytokines following testosterone treatment. Although these changes were not statistically significant, they align with the observed reductions in proinflammatory CD4^+^ T cell subsets, suggesting that testosterone may affect not only T cell frequencies but also their functional cytokine secretion profiles. These findings warrant further exploration to establish the robustness of these effects.

Mechanistically, it is assumed that testosterone modulates immune function mostly via the classical AR receptor, which is known to be expressed in various organs as well as most cells of the immune system, including peripheral T cells (44–47). In recent cancer studies, a central role has been described for AR signaling in CD8^+^ T cells. Activated CD8^+^ T cells showed increased AR expression, and AR signaling was central for the transition from stem cell-like CD8^+^T cells to terminally exhausted CD8^+^T cells (48, 49). For CD4^+^ T cells, a functional androgen response element within the Foxp3 locus has been described. Stimulation with androgens led to increased Foxp3 expression in human T cells from cis women, and the binding of AR was linked to changes in the acetylation status of histone H4 (50). More recently, it has been shown that AR signaling maintained the suppressive capacity of Treg in vitro; AR-mediated signaling in lung epithelial cells attenuated IL-33 release and thereby limited the differentiation toward ST2^+^ Treg, which are known to increase allergic airway inflammation (51). So far, most data on the use of testosterone in humans as a therapeutic agent are derived from randomized controlled trials investigating the role of testosterone supplementation for the treatment of hypoactive sexual desire disorders (4). In metastatic castration-resistant prostate cancers, AR signaling was shown to promote T cell exhaustion, and the signature of human CD8^+^ T cells was associated with increased intrinsic AR activity, while AR blockade could promote the response to anti–PD-1 immune checkpoint blockade (48).

In the context of autoimmune diseases, most studies were performed in translational animal models. Androgen treatment reduced the severity of EAE in a mouse model of MS and led to improved survival in male lupus NZB/NZW F1 mice (52, 53). In a nonobese diabetic (NOD) mouse model for type 1 diabetes (T1D), castration increased male T1D incidence and androgen treatment conferred protection to females, while in the NZB/W mouse model of lupus, testosterone was shown to delay or prevent murine lupus (54, 55). However, applying testosterone (150 μg) patches versus placebo to women with baseline mild-to-moderate SLE disease activity for 12 weeks was safe, but it did not significantly affect disease activity (56). Within our study, we can now report data from a single trans man who presented with AILD and who displayed substantially improved disease activity during GAHT. The in-depth analysis of T cells presumably activated before the start of GAHT revealed profound effects of testosterone therapy on CD4^+^ T cell activation status. This case highlights that a more detailed understanding of the androgen signaling pathway for T cell function in autoimmunity may reveal novel therapeutic targets in the future.

Our study has several strengths and limitations, including the limited number of trans men analyzed with single cell RNA-Seq (scRNA-Seq). Although we observed correlations between serum testosterone concentrations and T cell–related cytokine levels, we cannot prove the direct effect of testosterone on human T cells in vivo. Although we observed shifts in T cell phenotypes during GAHT, testosterone was associated with only subtle changes in CD4^+^ T cell transcriptomes in the group of trans men analyzed. It should be noted, however, that these were healthy, young people with likely high proportions of resting T cells. By contrast, profound effects on CD4^+^ T cell states were observed in the trans man with AILD during GAHT. This unique case reflects the rare combination of 2 uncommon conditions. While these data provide valuable insights into androgen signaling and its therapeutic potential, data derived from a single case clearly limits the ability to draw general conclusions, emphasizing the need for further studies in human AILD. Furthermore, the potential aromatization of testosterone into estrogen is an important consideration. In our study, low expression levels of aromatase (CYP19A1) were detected in T cells (data not shown), and no increase in aromatase expression was observed during in vitro testosterone treatment (Supplemental Figure 4A). Additionally, ex vivo analysis of testosterone and estradiol concentrations, along with corresponding Th1/Th17/Treg levels, did not indicate significant alterations in estradiol levels, supporting the conclusion that the observed effects were primarily driven by testosterone.

To our knowledge, this is the first report on the effects of testosterone in AILD highlighting sex hormones as modulators of T cell state and disease activity. Future studies should investigate how the interaction of T cells with hepatic parenchymal cells is modulated by sex hormones. In addition, longitudinal studies should investigate the effects of GAHT in trans men and trans women in more detail and over a longer period of time in order to appreciate the effects sex hormones have on human immunity.

## Methods

### Sex as a biological variable.

Sex is a potentially important variable in this study and has been taken into account in the analysis of the human material, experimental work with animals, and their interpretation. Therefore, we clearly stated which sexes where analyzed in the respective experiments.

### Serum and blood analysis.

Serum was obtained by centrifugation at 2,000*g* for 10 minutes and frozen at –80°C until further analysis. Clinical chemistry was analyzed at the Department of Clinical Chemistry, University Medical Center Hamburg-Eppendorf (UKE). Quantitative measurement of serum hormone levels was performed using MassChrom Steroids LC-MS/MS Assays (Chromsystems) by UPLC-ESI-MS (LCMS-8060, Shimadzu). The steroid measurements were carried out at the University Hospital Schleswig-Holstein, Campus Kiel (UKSH) in the pediatric endocrinology laboratory at the Department of Pediatrics and Adolescent Medicine I. The laboratory is accredited according to the Deutsches Institut für Normung/European Standard/International Standards Organization (DIN-EN-ISO 15189). Cytokine and chemokine levels were determined by flow cytometric-based LEGENDplex assay kits, specifically the HU Th Cytokine panel (12-plex) and HU Proinflammatory Chemokine panel 1 (13-plex) (both BioLegend), or ELISA measurement for TNF and IFN-γ (both Peprotech) according to manufacturer protocols.

### Immunophenotyping.

Heparinized whole blood was stained for immunophenotyping with different antibody cocktails; antibodies are listed in Supplemental Table 6. For the analysis of intracellular cytokines, whole blood samples were stimulated with PMA (250 ng/mL) and ionomycin (5 μg/mL, both Sigma-Aldrich) in the presence of GolgiPlug (1 μg/mL, BD Biosciences) for 4 hours. Surface antibodies were stained for 30 minutes at room temperature. Erythrocyte lysis and fixation were performed using RBC Lysis/Fixation solution (BioLegend) for 10 minutes followed by 2 subsequent washing steps with PBS. Stained cells were suspended in PBS containing 2% FCS and 0.01% NaN_3_. Samples were measured on the BD LSR Fortessa (BD Biosciences). Analysis were performed using FlowJo v10 (BD Biosciences) and the R Statistical Software (v4.1.2; R Core Team 2021) using the SPADE-Viz R package to analyze and integrate SPADE results (24). Gating strategies for identifying T cell subsets, including Th1, Th17, Treg, and other populations, are illustrated in Supplemental Figure 6, and the corresponding antibody information is detailed in Supplemental Table 6. For Supplemental Figure 4B, cryopreserved PBMCs from healthy trans men at baseline and the 6-month time point were thawed and stimulated with PMA (50 ng/mL), ionomycin (1 ng/mL), and Brefeldin A (1:100, Thermo Fisher Scientific) for 4 hours at 37°C. Surface antibodies were stained for 30 minutes at 4°C, followed by intracellular staining for 1 hour at room temperature using the eBioscience Foxp3/Transcription Factor Staining Buffer Set (Thermo Fisher Scientific). Analysis was performed using FlowJo v10 (BD Biosciences).

### Isolation of CD4^+^ T_NAIVE_ from PBMCs.

Blood samples were collected in EDTA tubes and stored overnight at 4°C. PBMCs were isolated by density gradient centrifugation at 600*g* (Ficoll-Paque PLUS, Cytiva). CD4^+^ T_N_ cells were isolated from PBMCs using the Pan T cell Kit or the Naive CD4^+^ T Cell Isolation Kit II, human (both Miltenyi Biotec), and purity was determined by flow cytometry (CD4^+^CD197^+^CD45RA^+^ cells). Only samples with a purity of ≥ 90% CD4+ TNAIVE cells (T_N_) of total CD4^+^ were used for further experiments.

### In vitro human T cell differentiation and stimulation.

Isolated Pan T cell or CD4^+^ T cells were cultured in the presence of anti-CD3 (2 μg/mL, clone OKT3, catalog 317353, Biolegend) and anti-CD28 (2 μg/mL, clone S20013F.REcH4, catalog 350902, Biolegend) in RPMI medium, supplemented with 10% charcoal-stripped FCS and 1% penicillin/streptomycin (Thermo Fisher Scientific). For Th1 differentiation, anti–IL-4 (2.5 μg/mL, clone REA8995, catalog130-124-309, Miltenyi Biotec), IL-2 (100 U/mL, R&D Systems, USA), and IL-12 (375 U/mL, Miltenyi Biotec) were supplemented, and cells were analyzed on day 6 using flow cytometry. Staining was performed using the eBioscience Foxp3/Transcription Factor Staining Buffer Set (eBioscience). For androgen treatment, different testosterone concentrations (3, 30, and 150 ng/mL) (T1500-1G, Sigma-Aldrich) and an ethanol dissolvent control (Th. Geyer) corresponding to the highest testosterone concentration were supplemented. Supernatants were harvested 24 hours after stimulation, and IFN-γ and TNF levels were analyzed using ELISA (both R&D Systems) according to the manufacturer instructions.

### RNA isolation and qPCR.

Total RNA was isolated with the NucleoSpin RNA Kit (Macherey-Nagel), and cDNA was reverse transcribed using the high-capacity cDNA reverse transcription kit (Thermo Fisher Scientific) according to the manufacturer’s instructions.

For quantitative PCR (qPCR) analysis, mRNA expression of various genes listed in Supplemental Table 7 was measured using TaqMan Fast Advanced Master Mix and TaqMan Gene Expression Assays (Thermo Fisher Scientific). Target gene expression was normalized to HPRT house keeper gene expression, and the fold-induction was quantified by normalization to control groups using the ΔΔCT method.

### FACS and antibody staining for sequencing.

Peripheral blood CD3^+^ T cells were isolated and sorted by FACS from previously cryopreserved PBMCs. Previously frozen cells were thawed and diluted with 50 mL of RPMI containing 10% FCS and 1% penicillin/streptomycin. After centrifugation at 400*g*, cells were washed with PBS and then stained with BV650-conjugated anti-CD3 (BioLegend) antibody at a 1:200 dilution in combination with live/dead staining using Fixable Viability Dye eFluor 506 (Thermo Fisher Scientific) at a dilution of 1:2,000. Additionally, cells were stained with an individual Total Seq C antibody cocktail (Supplemental Table 8). Staining of cells was performed for 30 minutes on ice after washing the cells. Cells were sorted on the BD Aria Fusion, and 20,000 cells were used for the Chromium Single-Cell platform (10X Genomics).

### Preparation of scRNA-Seq and CITE-Seq libraries.

The scRNA-Seq library was prepared using the 10X Chromium Next GEM Single Cell 5′ Library and Gel Bead Kit v1.1 according to the manufacturer’s instructions. Cells sorted by FACS were washed once with PBS containing 0.04% bovine serum albumin (BSA) and resuspended in PBS. In total, 20,000 cells were used for GEM generation through the 10X Chromium Controller using the Chromium Next GEM Chip K Single Cell Kit (10X Genomics). Briefly, droplet preparation was followed by reverse transcription and cell barcoding, the emulsions were resolved, and cDNA was purified using Dynabeads MyOne SILANE (Thermo Fisher Scientific) followed by PCR amplification with additional use of a primer for amplification of ADTs. Amplified cDNA was then used for construction of 5′ gene expression library, V(D)J-library, and ADT library using the dual index strategy following the manufacturer’s instructions. Quality control and quantification of the generated libraries was conducted using a 2100 Bioanalyzer Instrument (Agilent Technologies) and a QuBit 3.0 Fluorometer (Thermo Fisher Scientific), respectively.

### RNA-Sequencing.

The libraries were sequenced on an Illumina NovaSeq6000 to a minimum sequencing depth of 20,000 reads per cell for the gene expression library as well as a minimum sequencing depth of 5,000 reads for TCR and ADT libraries using read lengths of 100 bp read 1 (26 cycles), 8 bp i5 index (10 cycles), 8 bp i7 index (10 cycles), and 100 bp read 2 (90 cycles).

### scRNA-Seq data analysis.

Sequencing reads from the peripheral blood T cells were processed using Cell Ranger (version 4.0.0 10X Genomics) and the GRCh38-2020-A reference genome provided by 10X Genomics. The sequencing reads obtained from the transgender man AIH patient were processed using Cell Ranger version 7.0.1. SoupX (version 1.6.2), and scrublet (version 0.2.3) was applied to the individual count matrices produced by Cell Ranger to remove ambient RNA and doublet cells (57, 58). Additional data processing steps were performed with Seurat (version 4.0.0). Cells with a UMI count between 1,000 and 9,000 (20,000 for the AIH data) and a gene count between 500 and 2,800 (5,200 for the AIH data) genes as well as a mitochondrial gene expression percentage below 6% were kept for further analysis (57). The filtered expression data were normalized, scaled, and reduced in dimensionality by PCA following the Seurat default workflow. The peripheral blood T cells were integrated using Seurat’s Anchor Integration while the AIH T cells were integrated using Harmony (58). Following the data integration, Louvain clustering with a resolution of 0.9 (0.8 for the AIH cells) was performed. The resulting clusters were annotated according to their DEGs compared with all other cells. DEGs within clusters between time points were calculated by pseudobulk comparison using the Libra package (version 1.0.0) (59). ORA was performed with the enricher function from clusterProfiler (version 4.8.1) and the Hallmark gene sets provided by MSigDB (60,61). Fate mapping analysis within the CD4^+^ subset of the peripheral blood T cells was conducted with CellRank (1.5.1) on the PCA of the integrated data but separate for both time points. Fate probabilities were assessed for CellRank’s connectivity and pseudotime kernel, resulting in similar differentiation tendencies.

### Mice.

AR^fl/fl^ (B6.129S1-Artm2.1Reb/J) were obtained from The Jackson Laboratory and Lck^Cre^ mice [B6.Cg-Tg(Lck-icre)3779Nik/J] were provided by Samuel Huber (UKE). Both strains are bred on C57BL6/J background. AR^fl/fl^ Lck^Cre^ mice were generated by crossbreeding, and Cre-WT littermates were used as controls. All mice were bred and housed under specific pathogen–free conditions with 12-hour light/dark cycles at the animal care facility of the University Medical Center Hamburg-Eppendorf with access to standard chow diet (1318 rodent diet, Altromin) and water available ad libitum.

### Murine cell cultivation and stimulation.

Spleen cells were isolated as previously described. Isolated lymphocytes were cultivated in TexMACS (Miltenyi Biotec) supplemented with 1% penicillin/streptomycin in flat bottom 96-well plates. Cells were seeded at 5 × 10^5^ cells per well and stimulated for 24 hours using anti-CD3 (clone 145-2C11, catalog 100339, Biolegend) and anti-CD28 (clone D665.Rec, catalog 117003, Biolegend) (each 2 μg/mL, BD Biosciences). For proliferation assays, intracellular staining of isolated cells with Celltrace (Thermo Fisher Scientific) was performed according to the manufacturers’ instructions prior to TCR-specific stimulation, harvested on consecutive days, and analyzed using flow cytometry. For Th1 differentiation, anti–mouse IL-4 (clone 11B11, catalog 504101, Biolegend) (10 μg/mL), murine IL-2 (10 ng/mL), and murine IL-12 (10 ng/mL) were supplemented. For Th17 differentiation, murine IL-6 (20 ng/mL), murine IL-23 (10 ng/mL), murine IL-1β (10 ng/mL), human TGF-β (2 ng/mL), anti–mouse IL-4 (10 μg/mL), anti–mouse IL-2 (clone Jes6-1A12, catalog 503701, Biolegend) (10 μg/mL), and anti–mouse IFN-γ (10 μg/mL) were supplemented. For Treg differentiation, human TGF-β (2 ng/mL) was supplemented. Cells and supernatants were harvested on day 4 and analyzed by flow cytometry and ELISA. Cell staining was performed using the eBioscience Foxp3/Transcription Factor Staining Buffer Set (eBioscience). Supernatants were analyzed using ELISA for IL-17A, IL-22 and IFNgamma (both R&D Systems) according to the according to the manufacturer’s instructions.

### Clinical data.

PBC was diagnosed by the current criteria according to the European Association for the Study of the Liver (EASL) guidelines (20). Characteristics of healthy individuals and individuals with PBC can be found in Supplemental Table 1. Detailed information on the people analyzed in Figure 1D is presented in Supplemental Table 2, while Supplemental Table 1 corresponds to people included in Figure 1, A–C, E, and F. Female patients between the age of 37 and 69 years were selected after exclusion of advanced fibrosis (liver stiffness as measured by fibroscan > 11 kPa) or elevated serum bilirubin levels (>1.0 mg/dL). Two of the 24 female individuals with PBC were under immunosuppressive medication as indicated in Supplemental Table 1. Hormone levels of these people, derived from the same cohort and time points, are shown in Supplemental Figure 1. Postmenopausal status was queried, and the number of postmenopausal women included did not differ between the healthy control and PBC cohorts (Supplemental Figure 3D). Clinical data were obtained from electronic health records. Sex- and age-matched healthy blood donors were used as controls (HC). Fresh blood samples from trans men receiving GAHT were collected via the Institute for Sex Research, Sexual Medicine and Forensic Psychiatry, University Medical Center Hamburg-Eppendorf (UKE). Characteristics of the trans men cohort are summarized in Supplemental Tables 3–5.

### Statistics.

Statistical analysis was performed with GraphPad Prism (Version 10.2.3.403) and R. Data are presented as mean ± SD. Differences between 2 groups were assessed for statistical significance using one-way ANOVA, one-way ANOVA with correction for multiple comparison, or the Mann-Whitney *U* test, as indicated in the figure legends. Comparisons between more than 2 groups were performed by ordinary 1-way ANOVA test and Tukey’s post hoc test. *P* < 0.05 was considered significant.

### Study approval.

Fresh blood samples from cis women with PBC were collected via the YAEL Center for Autoimmune Liver Diseases, I. Department of Medicine, UKE. All donors participating in this study provided written informed consent according to the ethical guidelines of the IRB of the medical faculty of the University of Hamburg (PV5982, PV4081 and PV5473). Animal care was in accordance with the governmental and institutional guidelines and all experiments complied with the ARRIVE guidelines (62) and were approved by the local review board of the State of Hamburg, Germany (ORG 979 and ORG1119).

### Data availability.

Data of the single-cell sequences are available under ArrayExpress Accession ID E-MTAB-14141. For more details on the material and methods used, please contact the corresponding authors. Values for all data points in graphs are reported in the Supporting Data Values file.

## Author contributions

LH, NW, DS, and CS conceptualized the study and wrote the manuscript. LH, NW, and DS performed and analyzed experiments. LH and NW contributed equally to this work. DS and CS equally supervised the project. VH, CC, JM, DL, S Stein, FM, S Steinman, TP, JK, JF, JS, and AEK performed and analyzed experiments. PMH, SB, MA, SH, and AWL critically revised the manuscript for important intellectual content. All authors reviewed and edited the manuscript.

## Supplementary Material

Supplemental data

Unedited blot and gel images

Supporting data values

## Version 1 04/22/2025

Electronic publication
