# Supplementary material for: Testosterone affects female CD4+ T cells in healthy individuals and autoimmune liver diseases
Source: JCI Insight. 2025 Apr 22;10(8):e184544. doi: 10.1172/jci.insight.184544 (PMC12016935; doi:10.1172/jci.insight.184544)
Supplement: Supplemental data [file jciinsight-10-184544-s206.pdf]

Supplementary data:

Title: Testosterone affects female $CD4^+$ T cells in healthy individuals and autoimmune liver diseases

Authors: Lara Henze[1#], Nico Will[1#], Dakyung Lee[1], Victor Haas[1], Christian Casar[1,2], Jasper Meyer[1], Stephanie Stein[1], Franziska Mangler[1], Silja Steinmann[1], Tobias Poch[1], Jenny Krause[1], Johannes Fuss[3], Johanna Schröder[4], Alexandra E. Kulle[5], Paul-Martin Holterhus[5], Stefan Bonn[6,7], Marcus Altfeld[8], Samuel Huber[1,9], Ansgar W. Lohse[1,9], Dorothee Schwinge[1]*, Christoph Schramm[1,9,10]*

Affiliations:

[1] I. Department of Medicine, University Medical Center Hamburg-Eppendorf; Hamburg, Germany

[2] Bioinformatics Core, University Medical Center Hamburg-Eppendorf; Hamburg, Germany

[3] Institute of Forensic Psychiatry and Sex Research, Center for Translational Neuro- and Behavioral Sciences, University of Duisburg-Essen, Essen, Germany

[4] Institute for Clinical Psychology and Psychotherapy, Department for Psychology, Medical School Hamburg, Hamburg, Germany

[5] Divison of Pediatric Endocrinology and Diabetes, Department of Children and Adolescent Medicine, University Hospital Schleswig-Holstein, Campus Kiel, Kiel, Germany

[6] Institute of Medical Systems Biology, University Medical Center Hamburg-Eppendorf, Hamburg, Germany.

[7]Center for Biomedical AI, University Medical Center Hamburg-Eppendorf, Hamburg, Germany.

[8] Research Department Virus Immunology, Leibniz Institute of Virology, Hamburg, Germany.

[9] Hamburg Center for Translational Immunology, University Medical Center Hamburg-Eppendorf; Hamburg, Germany

[10] Martin Zeitz Center for Rare Diseases and, University Medical Center Hamburg-Eppendorf; Hamburg, Germany

[#]equally contributing authors

*equally shared senior authorship

Table of contents

Supplemental Figure 1 3

Supplemental Figure 2 4

Supplemental Figure 3 5

Supplemental Figure 4 6

Supplemental Figure 5 7

Supplemental Figure 6 8

Supplemental Table 1-2 Characteristics of the study cohort 9-10

Supplemental Table 3 Characteristics of trans men cohort for immunophenotyping 11

Supplemental Table 4 Trans men cohort for CITE-Seq analysis 12

Supplemental Table 5 Trans man with AIH/PSC variant syndrome 13

Supplemental Table 6 Antibodies for flow cytometry and Western blot (human/mouse) 14-16

Supplemental Table 7 TaqMan® Gene expression probes 17

Supplemental Table 8 Antibodies for CITE-Seq analysis 18

Supplementary data

Supplemental Figure 1:

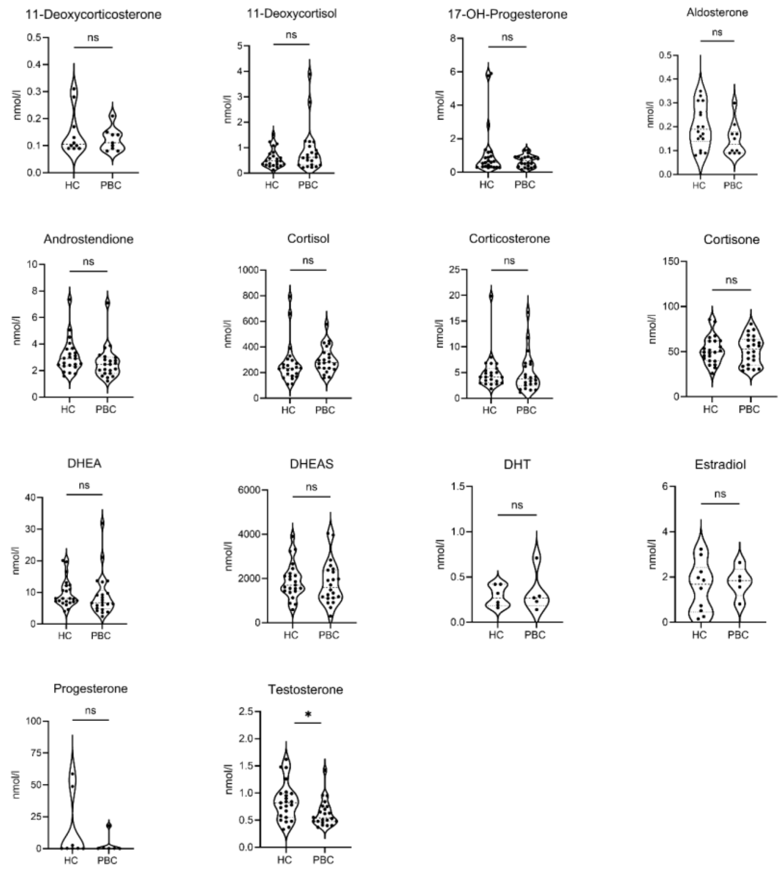

Supplemental Figure 1 Serum hormone profiles in cis women with PBC and age-matched controls

Quantitative analysis of 14 different serum sex hormone levels analyzed using liquid chromatography with tandem mass spectrometry (LC-MS/MS) in sera of women with PBC (n=27) compared to age-matched female healthy controls (n=23).

Supplemental Figure 2

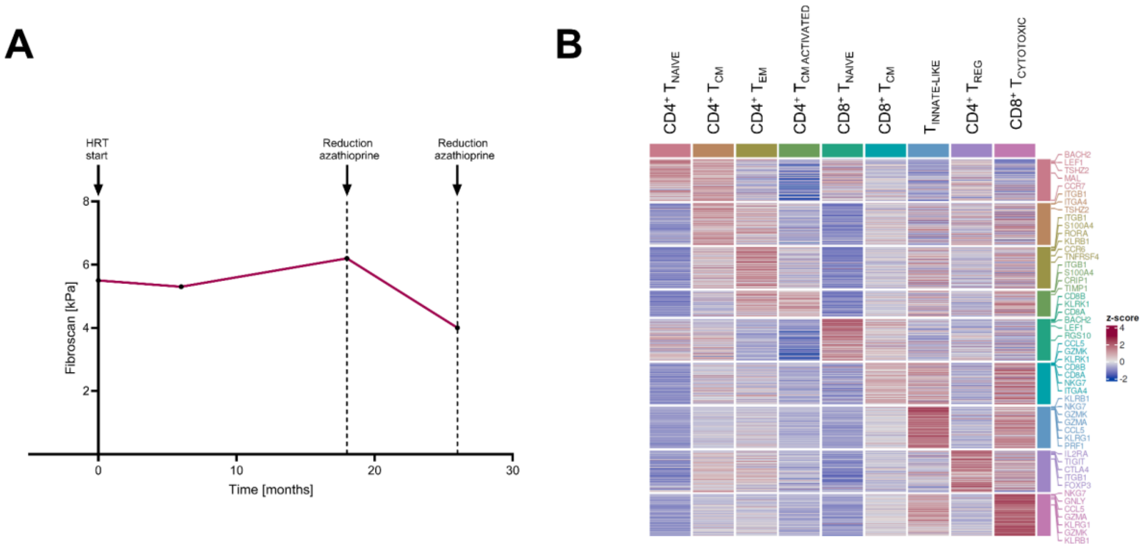

Supplemental Figure 2 Data from a single trans man with AIH/PSC variant syndrome receiving GAHT

(A) Improved liver stiffness during GAHT as determined by FibroScan® measurement. (B) Heatmap of signature differentially expressed genes (DEG) of each cell cluster obtained from 17,959 peripheral blood $CD3^+$ T cells at BL and 6M time point analyzed by CITE-Seq.

Supplemental Figure 3

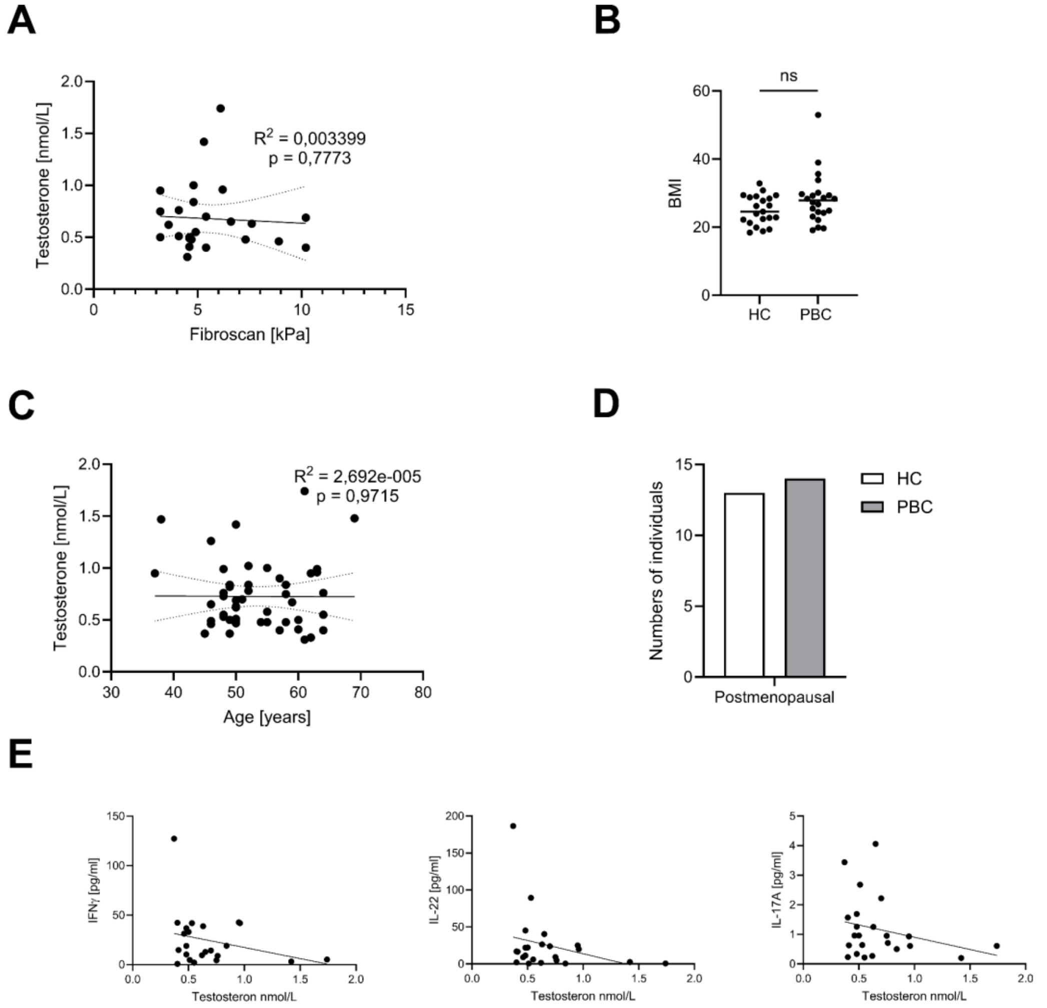

Supplemental Figure 3 Testosterone levels do not correlate with clinical and demographic parameters in healthy individuals and people with PBC

(A) Correlation between testosterone levels and liver stiffness as measured by FibroScan (kPa) in women with PBC. (B) Comparison of BMI between HC and PBC cohorts. (C) Correlation between testosterone levels and age in HC and PBC. None of the analyses revealed significant associations. (D) Number of individuals included in hormone analysis, all of whom are known to be postmenopausal. Numbers are comparable between HC and PBC. (E) Testosterone levels correlation to key cytokines in women with PBC ($p_{IFN\gamma}$= 0.19, $p_{IL\text{-}22}$= 0.17, $p_{IL\text{-}17A}$= 0.22).

Supplemental Figure 4

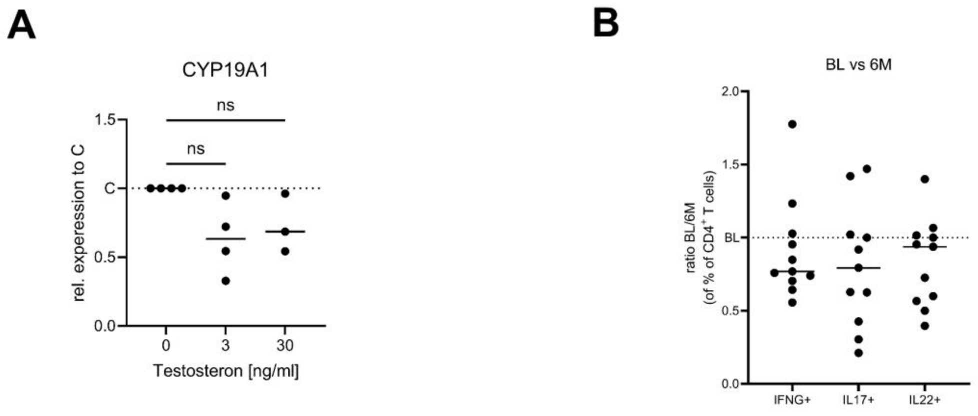

Supplemental Figure 4 Aromatase (CYP19A1) expression in T cells and cytokine production of T cells from the trans men under GAHT

(A) Relative expression of CYP19A1 in T cells during in vitro treatment with increasing concentrations of testosterone (0 ng/ml, 3 ng/ml, and 30 ng/ml). No significant increase in CYP19A1 expression was observed. (B) Ratios of baseline (BL) to 6-month (6M) levels of IFNy^{+}, IL-17^{+}, and IL-22^{+} CD4^{+} T cells derived from ex vivo analysis of PMA/Iono stimulated peripheral blood T cells.

Supplemental Figure 5

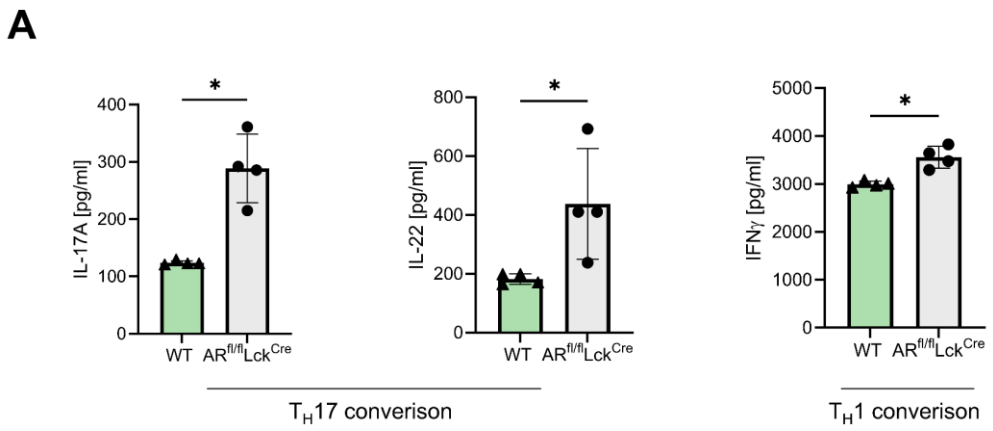

Supplemental Figure 5 Cytokine levels of murine derived T cells after in vitro differentiation

(A) Cytokine levels determined by ELISA in supernatants of in vitro differentiated $CD4^+$ T cells isolated from WT and AR deficient mice. Significant increases in IL-17A and IL-22 under T_H17 conversion and IFNγ under T_H1 conversion conditions were observed in AR deficient mice compared to WT controls (n=4 mice per group).

Supplemental Figure 6

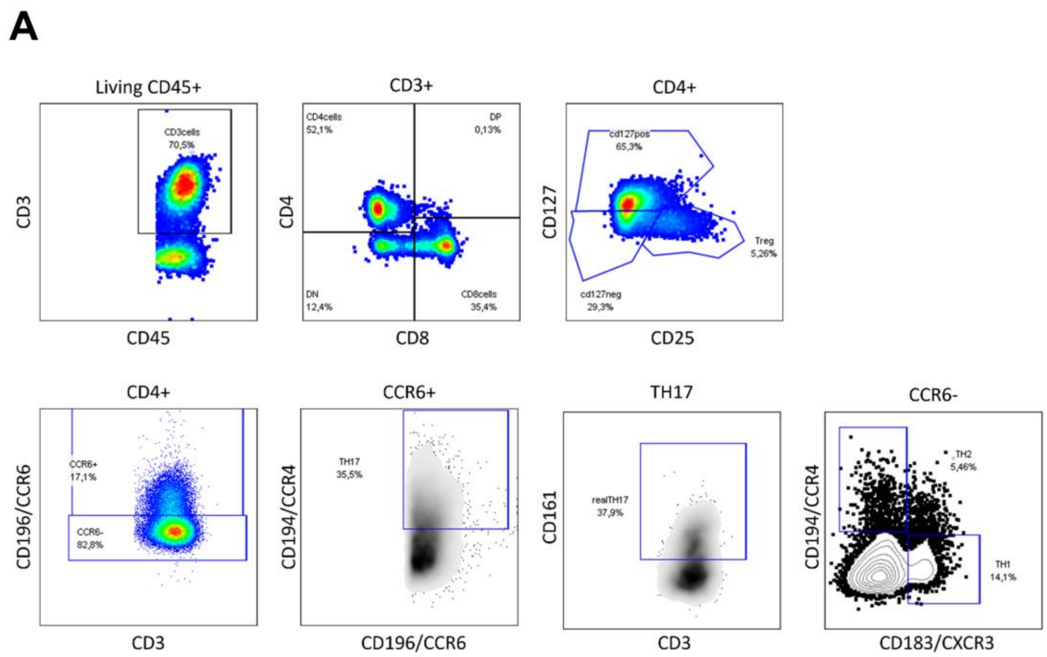

Supplemental Figure 6 Gating strategy for flow cytometry-based analysis of T cell subsets

(A) Gating strategy for flow cytometry-based ex vivo analysis of CD4^{+} T cell subsets, including T_{REG}, $T_{H}17$, $T_{H}1$, and $T_{H}2$ subsets presented in Figure 1C and Figures 3B-D.

Supplemental Table 1 Characteristics of the study cohort of Figure 1

Study group	Number	Age [yr]	BMI	WHR	Fibroscan [kPa]	ALT [U/L]	ALP [U/L]	Total BR [mg/dL]	UDCA	Immunosuppressive medication	Concomitant diseases	AMA (titer)	ANA (titer)	ASMA (titer)	IgG [g/L]
HC	23	52 ± 7.88	25 ± 4.3	0.81 ± 0.05	//	//	//	//	//	//	3: Glaucoma [1], Hashimoto's thyroiditis [1], Neurodermitis [1]	//	//	//	//
PBC	24	53 ± 6.71	27 ± 7.7	0.88 ± 0.10	4.85 ± 1.9	30 ± 15.35	112 ± 68.47	0.45 ± 0.25	20	2: Budesonide Inhalation [1], Hydroxychloroquine [1]	14: Arterial Hypertension [8], Hashimoto's Thyroiditis [3], Sicca/Sjögren Syndrome [3], Raynaud's Syndorme [2], Nephrolithiasis [2], Atopic Dermatitis [2], Lichen Planus [1], COPD [1], Astma bronchiale [1]	neg [4], 1:2560 [4], 1:320 [4], 1:5129 [4], 1:1280 [3], 1:640 [2], <1:5120 [1], 1:649 [1]	neg [7], 1:5120 [7], 1:160 [3], 1:5120 [1], <1:5120 [1], 1:1280 [2], 1:2560 [1], 1:80 [1]	neg [21], 1:160 [2]	11.9 ± 2.8

All participants of the table 1 and 2 are female. Data are presented as median values ± SD. Number in brackets [] are counts of participants with the medication/disease. ALT, alanine aminotransferase. ALP, alkaline phosphatase. HC, healthy control. PBC, primary biliary cholangitis. BMI, Body-Mass-Index. WHR, Waist–hip ratio. Total BR, Total Bilirubin. UDCA, Ursodeoxycholic acid. COPD, Chronic obstructive pulmonary disease.

Supplemental Table 2 Characteristics of the study cohort of Figure 1D

Study group	Number	Age [yr]	Fibroscan [kPa]	ALT [U/L]	ALP [U/L]	Total BR [mg/dL]	UDCA	Immunosuppressive medication	Concomitant diseases	AMA (titer)	ANA (titer)	ASMA (titer)	IgG [g/L]
HC	21	50 ± 10.3	//	//	//	//	//	//	2: Coagulation Factor V Disorder [1], Thalassemia minor [1]	//	//	//	//
PBC	16	53 ± 8.8	5.5 ± 2	29 ± 16	85 ± 88.1	0.5 ± 0.2	12	2: Azatioprine 150 mg [1], Methotrexate 12.5 mg/week [1]	12: Arterial hypertension [3], Chronic kidnes insufficiency stage 3 [1], Diabetic glomerulosclerosis [1], MASLD [1], Diabetes mellitus [1], Hypercholesterolesterolemia [2], MASH [1], Hashimoto's thyroiditis [1], Psoriatic arthritis [1], Hepatic steatisis grade I [1],	neg [7], 1:5120 [4], 1:320 [2], 1:1280 [1], 1:640 [1]	neg [4], 1:2560 [3], 1:320 [3], 1:1280 [2], 1:5120 [1], 1:160 [1]	neg [12], 1:160 [1], 1:80 [1]	10.8 ± 2.2

All participants of the table 1 and 2 are female. Data are presented as median values ± SD. Number in brackets [] are counts of participants with the medication/disease. ALT, alanine aminotransferase. ALP, alkaline phosphatase. HC, healthy control. PBC, primary biliary cholangitis. BMI, Body-Mass-Index. WHR, Waist–hip ratio. Total BR, Total Bilirubin. UDCA, Ursodeoxycholic acid. MASLD, metabolic dysfunction associated steatotic liver disease. MASH, Metabolic Dysfunction Associated Steatohepatitis.

Supplemental Table 3 Characteristics of trans men cohort for immunophenotyping

Study group	Age at BL [range]	6M (mean months after GAHT start)	GAHT number: testosterone injections	GAHT number: testosterone gel	additional GnRH-Analoga	Concomitant diseases	Medication
FTM	23 ± 11.1 [19-56]	6 ± 0.8	18 (180-250mg/ 4 weeks or 1000mg/ 3 months)	7 (25-50mg daily or unknown)	1	Hypothyroidism [2], Depression [2], Schizophrenia [1], Autism Spectrum Disorder [1], Graves' Disease [1], Hashimoto's Thyroiditis [1], Alopecia Universalis [1]	Levothyroxine [5], Mebeverine [1], Sertraline [1], Valsartan [1], Citalopram [1], Perazine [1], Benazepril [1], Omeprazole [1], Mesalazine [1], Cetirizine [1], Venlafaxine [1], Cholecalciferol [1], Folic Acid [1]

Data are presented as median values ± SD. Number in brackets [] are counts of participants with the medication/disease. GAHT, Gender-Affirming Hormone Therapy.

Supplemental Table 4 Trans men cohort for single-cell sequencing CITE-Seq analysis

Study group	Number of participants	Age at BL	6M (mean months after GAHT start)	GAHT (testosterone injections)	Concomitant diseases	Medication
FTM	4	24.5 ± 2.9	6.3 ± 0.4	4 (180-250mg/ 4 weeks or 1000mg/ 3 months)	Autism Spectrum Disorder [1]	Levothyroxine [5], Sertraline [1]

Data are presented as median values ± SD. Number in brackets [] are counts of participants with the medication/disease. GAHT, Gender-Affirming Hormone Therapy.

Supplemental Table 5 Trans man with AIH/PSC variant syndrome

Study group	Timepoint	Age	GAHT	Disease	Medication	ALT (IU/l)	ALP (IU/l)	Fibroscan [kPa]
FTM-AILD	BL	20	testosterone gel	AIH/PSC variant syndrome	Azathioprine (125mg), UDCA (2x500mg), VitD 20000IE	29	96	5.5
FTM-AILD	6M	20	testosterone gel	AIH/PSC variant syndrome	Azathioprine (125mg), UDCA (2x500mg), VitD 20000IE	33	71	5.3
FTM-AILD	18M	21	testosterone gel	AIH/PSC variant syndrome	Azathioprine (75mg), UDCA (2x500mg), VitD 20000IE	28	59	6.2
FTM-AILD	26M	22	testosterone gel	AIH/PSC variant syndrome	Azathioprine (50mg), UDCA (2x500mg), VitD 20000IE, (further reduction of Azathioprine to 25mg initiated)	23	56	4

Supplemental Table 6 Antibodies for flow cytometry and Western blot (human/mouse)

Article	Species	Type	Fluorophor	Catalog number	Company
CD103	human	Antibody	BV605	350218	Biolegend, Germany
CD127	human	Antibody	BV650	351326	Biolegend, Germany
CD16	human	Antibody	APC-Cy7	302018	Biolegend, Germany
CD160	human	Antibody	PerCP-Cy5.5	341210	Biolegend, Germany
CD161	human	Antibody	BV605	339916	Biolegend, Germany
CD161	human	Antibody	PE	339904	Biolegend, Germany
CD183	human	Antibody	BV711	353732	Biolegend, Germany
CD194	human	Antibody	PE-Cy7	359410	Biolegend, Germany
CD196	human	Antibody	PerCP-Cy5.5	353406	Biolegend, Germany
CD197	human	Antibody	APC	353214	Biolegend, Germany
CD199	human	Antibody	APC	358908	Biolegend, Germany
CD223	human	Antibody	APC	369212	Biolegend, Germany
CD25	human	Antibody	BV421	302630	Biolegend, Germany
CD272	human	Antibody	BV421	344512	Biolegend, Germany
CD279	human	Antibody	BV605	329924	Biolegend, Germany
CD28	human	Antibody	PE-Cy7	302926	Biolegend, Germany
CD3	human	Antibody	BV650	317324	Biolegend, Germany
CD3	human	Antibody	PerCP-Cy5.5	317336	Biolegend, Germany
CD39	human	Antibody	PE-Cy7	328212	Biolegend, Germany
CD4	human	Antibody	AF700	317426	Biolegend, Germany
CD4	human	Antibody	PE-Dazzle594	300548	Biolegend, Germany
CD44	human	Antibody	PE	338808	Biolegend, Germany
CD45	human	Antibody	BV785	304048	Biolegend, Germany
CD45RA	human	Antibody	BV711	304138	Biolegend, Germany
CD49a	human	Antibody	PE-Cy7	328312	Biolegend, Germany
CD49b	human	Antibody	FITC	359306	Biolegend, Germany
CD49d/ Integrin-α4	human	Antibody	PE	304304	Biolegend, Germany

CD56	human	Antibody	BV421	318328	Biolegend, Germany
CD57	human	Antibody	PE-Dazzle594	359620	Biolegend, Germany
CD62L	human	Antibody	BV510	304844	Biolegend, Germany
CD69	human	Antibody	PerCP-Cy5.5	310926	Biolegend, Germany
CD73	human	Antibody	PE	344004	Biolegend, Germany
CD8	human	Antibody	PE-Dazzle594	300930	Biolegend, Germany
CD8	human	Antibody	AF700	300920	Biolegend, Germany
Foxp3	human	Antibody	AF647	320214	Biolegend, Germany
Granzyme B	human	Antibody	FITC	515403	Biolegend, Germany
HLA-DR	human	Antibody	FITC	307604	Biolegend, Germany
IFNγ	human	Antibody	APC-Cy7	502530	Biolegend, Germany
IL-10	human	Antibody	PE	501404	Biolegend, Germany
IL-17A	human	Antibody	BV711	512328	Biolegend, Germany
IL-4	human	Antibody	PE-Cy7	500824	Biolegend, Germany
TNFα	human	Antibody	BV650	502938	Biolegend, Germany
β7-integrin	human	Antibody	FITC	121010	Biolegend, Germany
CD3	mouse	Antibody	Pacific Blue	100214	Biolegend, Germany
CD3	mouse	Antibody	BV510	100234	Biolegend, Germany
CD4	mouse	Antibody	BV650	100546	Biolegend, Germany
CD8a	mouse	Antibody	PerCP	100732	Biolegend, Germany
CD11b	mouse	Antibody	BUV737	612800	BD Bioscience, Germany
CD11c	mouse	Antibody	PE-Dazzle594	117348	Biolegend, Germany
CD25	mouse	Antibody	AF700	102024	Biolegend, Germany
CD25	mouse	Antibody	BV711	102049	Biolegend, Germany
CD45	mouse	Antibody	BV750	103157	Biolegend, Germany
CD45R/B220	mouse	Antibody	PE-Cy5	103210	Biolegend, Germany
CD279 (PD-1)	mouse	Antibody	BV711	135231	Biolegend, Germany
F4/80	mouse	Antibody	APC-Fire810	123166	Biolegend, Germany
Ly-6C	mouse	Antibody	PE-Cy7	128018	Biolegend, Germany
Ly-6G	mouse	Antibody	Spark Blue550	127664	Biolegend, Germany

NK-1,1 (CD161)	mouse	Antibody	BV510	108738	Biolegend, Germany
TCR γ/δ	mouse	Antibody	APC-Fire750	118136	Biolegend, Germany
TCR γ/δ	mouse	Antibody	BV421	118120	Biolegend, Germany
Foxp3	mouse	Antibody	APC	17-5773-82	eBioscience, Germany
GATA3	mouse	Antibody	AF488	653807	Biolegend, Germany
RORγt	mouse	Antibody	BV421	562894	BD Bioscience, Germany
T-bet	mouse	Antibody	PE	12-5825-80	eBioscience, Germany
Granzyme B	mouse	Antibody	FITC	515403	Biolegend, Germany
IFNγ	mouse	Antibody	AF700	505824	Biolegend, Germany
IL-4	mouse	Antibody	V450	560701	BD Bioscience, Germany
IL-10	mouse	Antibody	BV605	505031	Biolegend, Germany
IL-17A	mouse	Antibody	APC	17-7177-81	eBioscience, Germany
TNFα	mouse	Antibody	PE-Cy7	506324	Biolegend, Germany
Androgen	mouse	Antibody		ab133273	Abcam, Germany
Beta-Actin	mouse	Antibody		sc-47778	Santa Cruz, Germany
CD45	human	Antibody	BUV805	612891	BD Bioscience, Germany
CD3	human	Antibody	BUV395	564001	BD Bioscience, Germany
CD4	human	Antibody	BUV 496	612936	BD Bioscience, Germany
CD8a	human	Antibody	BV510	301048	Biolegend, Germany
CCR6	human	Antibody	AF488	353414	Biolegend, Germany
CXCR3	human	Antibody	BV711	353732	Biolegend, Germany
CD25	human	Antibody	AF700	302622	Biolegend, Germany
CD127	human	Antibody	Pacific Blue	351306	Biolegend, Germany
CD39	human	Antibody	BV785	328239	Biolegend, Germany
CCR4	human	Antibody	PE-Cy7	359410	Biolegend, Germany
CD161	human	Antibody	BUV563	749223	BD Bioscience, Germany
L/D	human		Zombie NIR Fixable Viability Kit	423106	Biolegend, Germany
IFNγ	human	Antibody	BV650	502538	Biolegend, Germany
TNF	human	Antibody	PerCP	502924	Biolegend, Germany
IL-17A	human	Antibody	BV605	512326	Biolegend, Germany
FOXP3	human	Antibody	AF647	320214	Biolegend, Germany
IL10	human	Antibody	PE	16103272	Biolegend, Germany
IL22	human	Antibody	BUV737	367-729-42	Invitrogen, Germany

Supplemental Table 7 TaqMan® Gene expression probes

Gene	species	Catalog Number	Company
TNF	human	Hs01113624_g1	Thermofisher, Germany
IFN	human	Hs00989291_m1	Thermofisher, Germany
GAPDH	human	Hs02786624_g1	Thermofisher, Germany
Ar	mouse	Mm00442688_m1	Thermofisher, Germany
GAPDH	mouse	Mm99999915_g1	Thermofisher, Germany

Supplemental Table 8 Antibodies for CITE-Seq analysis

Article	species	Type	Catalog Number	C
C0032 CD154	human	TotalSeq™ C Antibody	399905	Biolegend, Germany
C0034 CD3	human	TotalSeq™ C Antibody	399905	Biolegend, Germany
C0046 CD8	human	TotalSeq™ C Antibody	399905	Biolegend, Germany
C0063 CD45RA	human	TotalSeq™ C Antibody	399905	Biolegend, Germany
C0071 CD194 (CCR4)	human	TotalSeq™ C Antibody	399905	Biolegend, Germany
C0072 CD4	human	TotalSeq™ C Antibody	399905	Biolegend, Germany
C0085 CD25	human	TotalSeq™ C Antibody	399905	Biolegend, Germany
C0088 CD279 (PD-1)	human	TotalSeq™ C Antibody	399905	Biolegend, Germany
C0139 TCR γ/δ	human	TotalSeq™ C Antibody	399905	Biolegend, Germany
C0140 CD183 (CXCR3)	human	TotalSeq™ C Antibody	399905	Biolegend, Germany
C0143 CD196 (CCR6)	human	TotalSeq™ C Antibody	399905	Biolegend, Germany
C0147 CD62L	human	TotalSeq™ C Antibody	399905	Biolegend, Germany
C0148 CD197 (CCR7)	human	TotalSeq™ C Antibody	399905	Biolegend, Germany
C0149 CD161	human	TotalSeq™ C Antibody	399905	Biolegend, Germany
C0168 CD57 Recombinant	human	TotalSeq™ C Antibody	399905	Biolegend, Germany
C0170 CD272 (BTLA)	human	TotalSeq™ C Antibody	399905	Biolegend, Germany
C0386 CD28	human	TotalSeq™ C Antibody	399905	Biolegend, Germany
C0390 CD127 (IL-7Rα)	human	TotalSeq™ C Antibody	399905	Biolegend, Germany
C0581 TCR Vα7.2	human	TotalSeq™ C Antibody	399905	Biolegend, Germany
C0584 TCR Vα24-Jα18	human	TotalSeq™ C Antibody	399905	Biolegend, Germany
