# Supplementary figures and images for: Testosterone affects female CD4+ T cells in healthy individuals and autoimmune liver diseases
Source: JCI Insight. 2025 Apr 22;10(8):e184544. doi: 10.1172/jci.insight.184544 (PMC12016935; doi:10.1172/jci.insight.184544)

Androgen receptor (AR)  
Abcam #ab133273

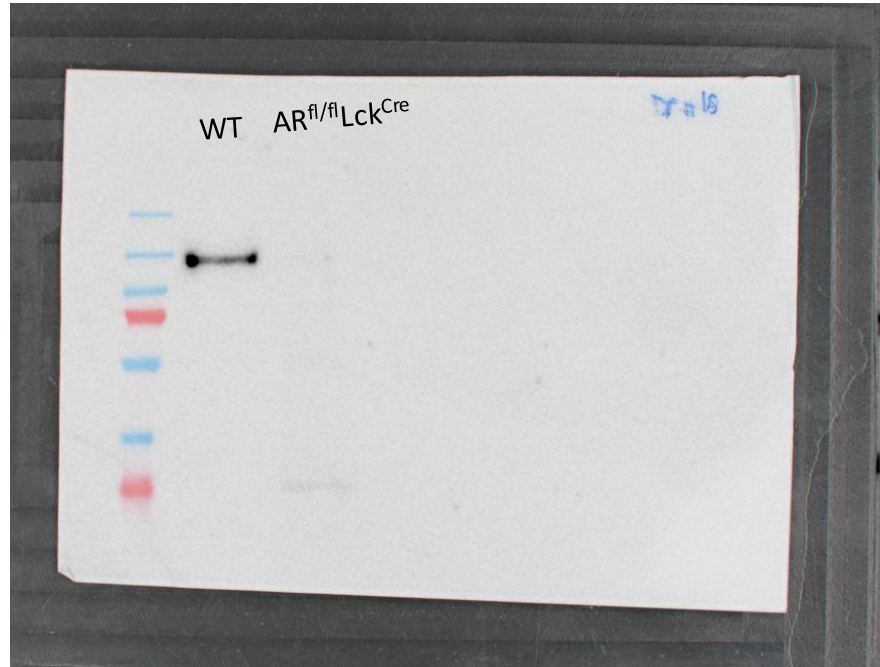

Beta actin  
Santacruz #sc-47778

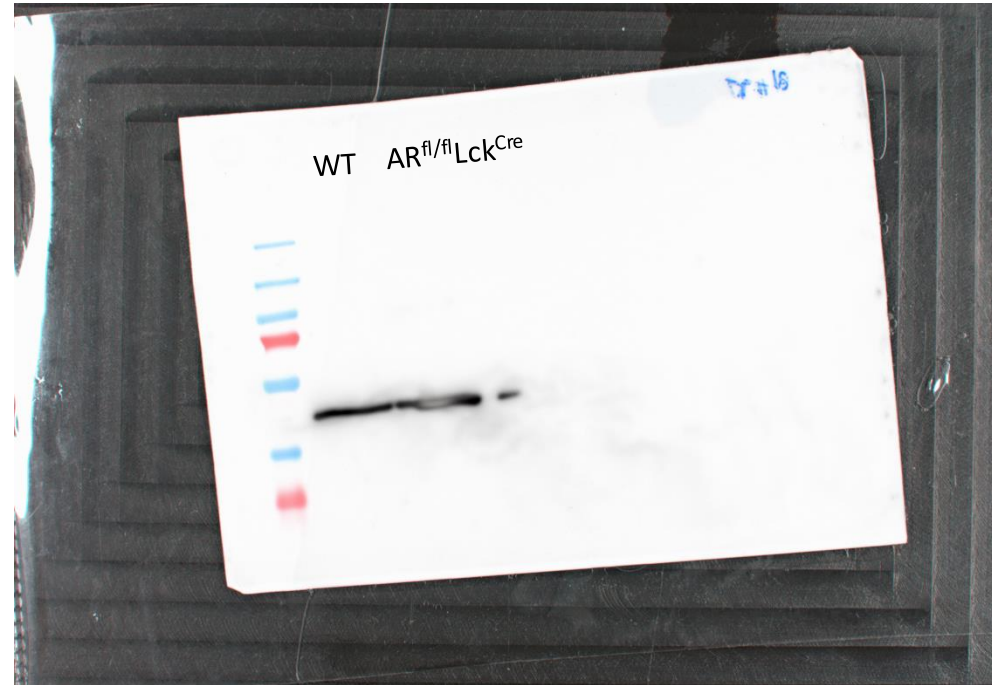

Supplement: Unedited blot and gel images [file jciinsight-10-184544-s207.pdf]
